# Type 2 Diabetes Mellitus, Oral Diabetic Medications, Insulin Therapy, and Overall Breast Cancer Risk

**DOI:** 10.1155/2013/181240

**Published:** 2013-01-17

**Authors:** Hala Ahmadieh, Sami T. Azar

**Affiliations:** Division of Endocrinology and Metabolism, Department of Internal Medicine, American University of Beirut Medical Center, Beirut, Lebanon

## Abstract

Breast cancer is among the most common cancers worldwide. Diabetes is an important chronic health problem associated with insulin resistance, increased insulin level, changes in growth hormones and factors, and activation of mitogen-activating protein kinase (MAPK) pathways, leading to an increased breast cancer risk. This paper looked at the epidemiologic studies of the association between type 2 diabetes and risk of breast cancer and its effect on overall cancer-specific survival. The combined evidence overall supported a modest association between type 2 diabetes and the risk of breast cancer, which was found to be more prevalent among postmenopausal women. Effect of oral diabetics and insulin therapy on breast cancer risk was also evaluated. It was found that metformin and thiazolidinones tended to have a protective role. Metformin therapy trials for its use as an adjuvant for breast cancer treatment are still ongoing. Sulfonylurea and insulin therapy were found to be mildly associated with increased overall cancers. No evidence or studies evaluated the association of DPPIV inhibitors and GLP 1 agonists with breast cancer risk because of their recent introduction into the management of diabetes.

## 1. Introduction

Breast cancer is among the most common cancers worldwide and is the second leading cause of cancer death for women in the United States, after lung cancer, with an estimated incidence of 226,870 cases and estimated deaths of 39,510 cases in the year of 2012. The National Cancer Institute also estimated that 1 in 8 women in the United States has the chance of developing invasive breast cancer throughout her lifetime [1, 2]. Diabetes is also a very common chronic health problem where it is currently estimated that 10% of women in the United States over the age of 20 have type 2 diabetes. Prevalence of diabetes has steadily increased since 1990. The 2010 CDC study projected that by 2050, as many as one of three US adults could have diabetes if the current trend continues [3, 4]. Association between diabetes and breast cancer has been noted where 16% of older breast cancer patients were found to suffer from diabetes, and this might have important public health implications. 

## 2. Pathogenesis

**Figure 1:**
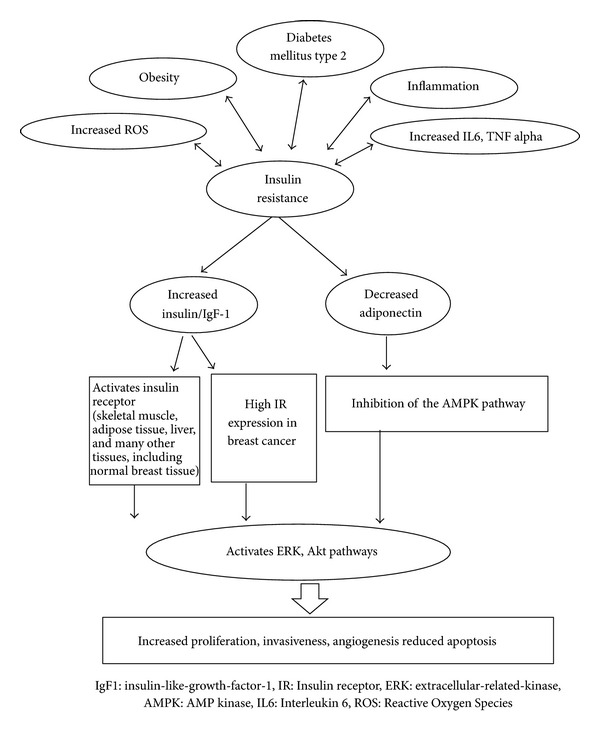
Link between type 2 diabetes, insulin resistance and increased breast cancer risk of development: pathophysiology. Link of diabetes mellitus type 2 with breast cancer: pathophysiology.

Different mechanisms contribute to the association between diabetes and breast cancer. Diabetes induces several changes in different hormonal systems including insulin, insulin-like growth factors, estrogen, and other growth factors, all of which may affect the risk for breast cancer development. As shown in Figure 1, type 2 diabetes mellitus is associated with insulin resistance, inflammation with increased inflammatory markers such as Interleukin 6 and increased reactive oxygen species with all of which being related to high insulin level, which in turn activates the insulin receptor, which is a tyrosine kinase receptor, expressed in skeletal muscle, adipose tissue, liver, and other tissues including normal breast tissue. Insulin receptor, once activated, will phosphorylate a number of intracellular proteins, leading to the activation of the extracellular signal-regulated kinase (ERK) cascade, one of the mitogen-activating protein kinase (MAPK) pathways, which increases mitogenesis and breast cancer risk. Insulin also suppresses IGF binding protein-1, thus increasing bioavailable IGF-1. Diabetes is also associated with decreased adiponectin plasma levels, which inhibits the AMP kinase (AMPK) and thus activates the ERK and Akt pathways leading to increased breast cancer risk [5, 6].

### 2.1. Association of Diabetes and Incidence of Breast Cancer

With regards to the complex association between type 2 diabetes and incidence of breast cancer, several prospective and case-control studies reported increased risk ratios for breast cancer among women with type 2 diabetes [7–14]. However, other studies found no association [15–25]. It is important to note that the above studies included small sample size, did not include potential confounders, and were underpowered to look at the real association. One of those studies, however, which was the Iowa Women's Study did adjust for BMI and waist-to-hip ratio and did not reveal an overall association between type 2 diabetes and breast cancer incidence [23]. The Nurses' Health Study, provided the largest population, had the longest followup that included a total of 116,488 female nurses, whose ages were 30–55 years old and who were free of cancer in 1976, and then followed up through 1996 for the occurrence of type 2 diabetes and through 1998 for the occurrence of incident invasive breast cancer, as verified by medical records and pathology reports. Women with type 2 diabetes were found to have a modestly elevated incidence of breast cancer (hazard ratio of 1.17; 95% CI 1.01–1.35) compared with women without diabetes, and this was independent of age, obesity, family history of breast cancer, history of benign breast disease, reproductive factors, physical activity, and alcohol consumption. This association was only apparent among postmenopausal women (1.16; 0.98–1.62) but not among premenopausal women (0.83; 0.48–1.42) and among women who had estrogen receptor-positive breast cancer (1.22; 1.01–1.47) [26]. Moreover a recent large meta-analysis reported that there were about a 20% increase in risk for both case-control and cohort studies [27]. The effect of self-reported diabetes on breast cancer incidence and the 5-year specific mortality was also evaluated in the Long Island Breast Cancer Study Project, which included 1,447 breast cancer cases and 1,453 controls from the National Death Index. This study noted an increased risk of breast cancer development among postmenopausal women with diabetes (OR = 1.35; 95% confidence interval (CI) = 0.99–1.85), as were those who were not of white race regardless of their menopausal status (OR = 3.89; 95% CI = 1.66–9.11) [28]. The recently published WHI clinical trials program involved 68,000 postmenopausal women who were observed prospectively; 11,290 of which had diabetes at study entry or later developed it during followup; 3,273 developed invasive breast cancer after study entry. This study, in contrary to the Nurses' Health Study, showed that breast cancer risk was not different in women with diabetes as compared with those without diabetes (hazard ratio (HR), 0.99; 95% CI, 0.85 to 1.14 for invasive disease; HR, 0.99; 95% CI 0.73 to 1.36 for ductal carcinoma in situ), after adjustment for obesity and physical inactivity [29].

### 2.2. Association between Diabetes and Breast Cancer Risk

 A cohort study within the UK General Practice Research Database found that diabetes was associated with 29% increased breast cancer risk (95% CI: 1.16–1.44), but the association was markedly attenuated when adjusted for age, region, and body mass index (BMI) (HR: 1.12; 95% CI: 0.98–1.29). This cohort also showed that women with breast cancer who had preexisting diabetes had an overall increased mortality of 49%, (95% CI: 1.17–1.88), as compared to breast cancer without diabetes and this persisted even after controlling for age, period, region, BMI, smoking, alcohol, and deprivation [30]. In another study, including 4,390 Asian patients with breast cancer, of which 341 (7.7%) presented with DM, the 5-year breast cancer survival and overall survival was significantly lower diabetics as compared to nondiabetics (BCS, 85% versus 91%; OS, 79% versus 90%), respectively. This persisted even after adjusting for all clinical variables and comorbidities [31]. In a retrospective study, including breast cancer patients who had undergone mastectomy and completed adjuvant chemotherapy from 1998 to 2010, median disease-free survival was found to be 81 months (95% CI, 61.6–100.4) in nondiabetic patients and 36 months (95% CI, 13.6–58.4) in diabetic patients (*P* < 0.001) [32]. On the other hand, in a retrospective analysis of 265 patients with advanced breast cancer, no difference in overall survival was observed between the diabetic and nondiabetic patients, but this study showed that the overall survival was greater in diabetic patients who had proper metabolic control as compared to those with poor metabolic control [33]. Moreover, in the Fremantle Diabetes Study (FDS), which was a community-based longitudinal observational study of 1426 subjects, 1294 of which had type 2 diabetes, it was found that diabetic men and women had similar risks of prostate and breast cancer like those of controls [34]. 

A recent meta-analysis showed that the relative risk for breast cancer in women with diabetes was 1.27 (95% confidence interval (CI), 1.16–1.39). Prospective studies showed a lower risk (SRR 1.23 (95% CI, 1.12–1.35)) as compared to retrospective studies (SRR 1.36 (95% CI, 1.13–1.63)). Type 1 diabetes, or diabetes in premenopausal women, was not associated with the risk of breast cancer (SRR 1.00 (95% CI, 0.74–1.35) SRR 0.86 (95% CI, 0.66–1.12), resp.). Studies adjusting for body mass index (BMI) showed lower estimates (SRR 1.16 (95% CI, 1.08–1.24)) as compared with those studies that were not adjusted for BMI (SRR 1.33 (95% CI, 1.18–1.51)). It was concluded that the risk of breast cancer in women with type 2 diabetes is increased by 27%, but decreased to 16% after the adjustment for BMI. No increased risk was seen for women at premenopausal ages or with type 1 diabetes [35]. Also, a recent case-control study that evaluated the risk of breast cancer risk in Uruguayan women was carried out between 2004 and 2009, including 912 women of ages between 23 and 69 years (367 new BC cases and 545 nonhospitalized, age-matched controls with a normal mammography). This study showed that a personal history of diabetes was positively associated to breast cancer risk (OR = 1.64, 95% CI 1.00–2.69), being higher among postmenopausal women (OR = 1.92, 95% CI 1.04–3.52) and even significantly more increased among postmenopausal women who are overweight and had dislypidemia (OR = 9.33, 95% CI 2.10–41.5) and high fat/muscle ratio (OR = 7.81, 95% CI 2.01–30.3) [36].

In a recent review of the association of diabetes, metabolic syndrome, and breast cancer risk, included were 26 studies, of which 10 were case-control studies, 3 of which directly looked at the association of diabetes and breast cancer [20, 37, 38], 14 were cohort studies [26, 39–42], 5 of which looked at the above association, and 2 were cross-sectional studies [43, 44]. This paper supported a modest association between type 2 diabetes and the risk of breast cancer, which appears to be more consistent among postmenopausal as compared to premenopausal women. It was proposed in this review that hyperinsulinemic state would suppress SHBG, hence increasing free available estrogen concentrations. In addition, IGF-I stimulates the production of androgens in the ovarian stroma, which displaces estrogens from SHBG. They also added that estradiol alters the expression of many components of the IGF-I system; where the ligand-bound estrogen receptor binds to and activates IGF-1R directly and as a result IGF-I signaling enhances estrogen receptor activation leading to the phosphorylation of the estrogen receptor. This leads to IGF-I and estrogen having a synergistic effects leading to proliferation and increasing breast cancer risk in the presence of the hyperinsulinemic state of type 2 diabetes [45, 46].

### 2.3. Diabetes Medications and Breast Cancer Risk

#### 2.3.1. Metformin Use and Breast Cancer Risk

Several observational studies suggested that metformin use decreases the incidence of several cancers overall [47–50]. For example, Evans and colleagues [47] reported a decreased risk of breast cancer in diabetics receiving metformin (versus those patients not on metformin), with the protective effect increasing with the increase in metformin exposure. However, findings regarding breast cancer and metformin use have been mixed. A recent meta-analysis which included seven independent observational studies supported a protective effect of metformin on breast cancer risk among postmenopausal women with diabetes (OR was 0.83). Stronger associations were noted with longer metformin use [51]. In addition, in the cohort study within the UK General Practice Research Database, it was found that metformin monotherapy had weaker association with breast cancer risk with a hazard ratio of 1.04 (95% CI: 0.79–1.37) as compared to sulfonylurea and insulin where the latter were more associated with breast cancer risk (HR: 1.33; 95% CI: 0.63–2.83) [30]. A case-control study also demonstrated a decreased risk of breast cancer in women who took metformin for several years as compared to short-term users [52]. Moreover, a nested case-control study of the Danish medical registry included 4323 type 2 diabetic perimenopausal or postmenopausal women and showed that those who used metformin for at least 1 year were less likely to be diagnosed with breast cancer as compared to those who did not use metformin. Moreover this association was not altered even after adjustment for obesity, diabetes complications, and other predictors of breast cancer [53]. 

As for the proposed protective effect of metformin, it is well known that metformin acts by increasing glucose uptake by skeletal muscle and thus reducing hyperglycemia by improving insulin sensitivity through the stimulation of the adenosine monophosphate-activated protein kinase (AMPK), leading to the suppression of gluconeogenesis, protein, and fatty acid synthesis resulting in partial metabolic normalization of hyperglycemia and insulin resistance [54, 55]. Stimulation of the AMPK inhibits mammalian target of rapamycin (mTOR)/ribosomal S6 kinase pathway. This would inhibit pathological cell cycle progression, cell growth, and angiogenesis [56, 57]. Moreover stimulation of AMPK by metformin led to decrease cell proliferation in both estrogen receptor alpha (ER alpha) negative and positive human breast cancer cell lines, in addition to the inhibition of aromatase expression in human breast adipose stromal cells [58, 59]. In vitro studies have also shown that metformin use reduces the proliferation of breast cancer cells [60]. The role of metformin as anti-breast cancer agent has also been attributed to its effect on immune system but this has to be further looked at in more details [61].

Recently, an epidemiological study of 2,529 women with breast cancer reported higher pathologic complete response in diabetic patients on neoadjuvant systemic therapy and receiving metformin (pCR 24%) as compared to diabetic patients not receiving metformin (pCR 8%) and nondiabetic patients not receiving metformin (pCR 16%) [62]. However metformin failed to significantly improve the estimated 3-year relapse-free survival rate in this study. Some prospective clinical trials have been completed in nondiabetic patients who received low doses of metformin (250 mg/day) and showed a reduction in the proliferative activity of colonic epithelium [63]. This led to ongoing studies involving neo-adjuvant metformin treatment of newly diagnosed breast cancer patients, which have also demonstrated that metformin has favorable effects on tumor cell proliferation and apoptosis [64, 65].

A currently ongoing phase III trial of metformin versus placebo in early-stage breast cancer, where nondiabetic women or men, younger than age 75 with newly diagnosed early-stage breast cancer, within the previous 12 months, and following their surgery to remove their tumor, will be randomly assigned to take metformin or placebo pills twice a day for 5 years. Participants in the trial may also receive adjuvant hormone and/or radiation therapy, but if chemotherapy (adjuvant or neoadjuvant) was given, it must have been completed prior to joining the study. This study will mainly monitor to see if metformin would improve disease-free survival, overall survival, and a number of other medical, biological, and quality-of-life endpoints [66].

Other 6 ongoing studies are currently done evaluating the efficacy and safety of treating cancer patients with the metformin. The European Institute of Oncology in Italy is currently planning a presurgical randomized, double-blind, placebo-controlled phase II trial in which 100 histologically confirmed that breast cancer patients not suitable for neoadjuvant therapy will be assigned randomly to either metformin (850 mg twice/daily or placebo until surgery with the aim of evaluating the activity of metformin on Ki67-measured tumor proliferation [67]. Also in Italy, two randomized clinical trial, one of them is the Plotina plan, aim to evaluate the effect of metformin on breast cancer as primary prevention in around 16,000 postmenopausal women, aged 45–74 years, where patients are being randomly assigned to the metformin treatment or placebo, and histologically confirmed invasive breast cancer diagnosed after recruitment to the trial (date at interview) and before the end of the last follow-up period. The results of the two trials will clarify the role of metformin as a chemopreventive agent [68].

Another phase II, randomized, open-label, multicentric clinical trial wants to evaluate HER2-related benefits of metformin as neoadjuvant chemotherapy with chemotherapy and trastuzumab in women diagnosed with HER2-positive primary breast cancer with the assessment of its effect on a 3-year disease free survival [69].

#### 2.3.2. Thiazolinediones and Breast Cancer

Three nested case-control studies, included 513 breast cancer patients as compared to 2557 controls, were used to evaluate the risk of breast, colon, and prostate cancers developing in patients exposed to thiazolidinediones (TZDs) as compared with other antidiabetic agents and they showed a neutral effect of TZDs on the likelihood of the development of cancers including colon, prostate, and breast cancers [70].

The therapeutic effects of troglitazone, in patients with refractory metastatic breast cancer to at least one chemotherapy regimen (ER negative tumors) or two hormonal regimens (ER positive tumors), were evaluated before it was withdrawn from the market following FDA warnings on hepatic toxicity. No objective responses were observed and it was found to have little apparent clinical value among patients with treatment-refractory metastatic breast cancer [71]. In a study of 1983 consecutive patients with HER2+ breast cancer treated between January 1 1998 and September 30 2010, it was shown that metformin (*P* = 0.041, HR = 0.52, 95% CI 0.28–0.97) and thiazolidinediones (*P* = 0.036; HR = 0.41, 95% CI 0.18–0.93) significantly lengthened survival and decreased breast cancer-specific mortality (*P* = 0.023, HR = 0.47, 95% CI 0.24–0.90 and *P* = 0.044, HR = 0.42, 95% CI 0.18–0.98, resp.) [72]. It was proposed that thiazolenediones has a protective role decreasing breast cancer risk through downregulating Wnt/Catenin Signaling, hence targeting abnormal breast cancer cells directly [73].

#### 2.3.3. Sulfonylurea and Breast Cancer

In a recent review, it was found that the first and second generation sulfonylureas, but not the third generation glimepiride, and glinides increased the risk of overall cancer, specifically hepatocellular cancer but it was less frequently associated with breast cancer, pancreatic cancer, or bladder cancer. It was also found that this increased but slightly less risk as compared to insulin supported the hypothesis that an increasing insulin level plays an important role in carcinogenesis [74]. No other studies looked at the particular association of sulfonylurea use and breast cancer risk.

#### 2.3.4. DPPIV Inhibitors and Breast Cancer Risk

Dipeptidyl peptidase-IV (DPP-IV) inhibitors, as well as glucagon-like peptide-1 (GLP-1) agonists, are relatively new medications used in the treatment of DM2 where GLP-1 agonists, exenatide, and liraglutide were first introduced in the USA in 2005 and sitaglipitin, first DPP IV inhibitor introduced in 2006. These therapies are effective in preserving *β*-cell mass by improving islet cell function through inhibiting apoptosis. 

Their relatively short-term use clinically does not permit any meaningful data on their malignancy risks. Sitagliptin did show increased pancreatic ductal hyperplasia in a small rodent model study which may predispose to pancreatic cancer risk although one short-term study involving human pancreatic cancer cell lines did not show this [75–77]. No data is available on the association of DPPIV inhibitors, GLP-1 agonists, and breast cancer risk due to their introduction recently in the diabetes management.

#### 2.3.5. Insulin Treatment and Breast Cancer

A recent meta-analysis, including 562,043 participants and 14,085 cases of cancer, was published assessing the risk of cancer during treatment with insulin. It showed that insulin treatment was associated with an increased risk of overall cancer (RR (95% CI) = 1.39 (1.14, 1.70)) especially with pancreatic cancer (RR (95% CI) = 4.78 (3.12, 7.32)) [78]. As for the association of insulin therapy with breast cancer, the UK's General Practice Research Database included a cohort of 15,227 women with type 2 diabetes, treated with insulin glargine (4,579 users) and matched with users of other insulins (10,648 users), and followed up till the first breast cancer diagnosis or until the end of December 2009, of which 246 developed breast cancer during the 8-year followup. It was shown that insulin glargine was not associated with an increased risk of breast cancer during the first 5 years of use. However, longer-term use may increase this risk, particularly in women with the longstanding use of insulin before starting insulin glargine [79]. Significant association of insulin therapy and breast cancer cases cannot be deduced due to the lack of substantial evidence. In the ORIGIN trial, which was primarily oriented at determining whether the use of insulin glargine, as compared to standard care, in patients with impaired fasting glucose or impaired glucose tolerance, would affect cardiovascular outcome, insulin glargine was not shown to have any significant increase in cancers (hazard ratio, 1.00; 95% CI, 0.88 to 1.13; *P* = 0.97). It is important to note that this study included 12,537 participants whose mean age was around 65 who had cardiovascular risk factors plus impaired fasting glucose, impaired glucose tolerance, or early diabetes. Insulin glargine was shown to have a neutral effect on cardiovascular outcomes and cancers [80].
